# Supplementary material for: Length-dependent accumulation of double-stranded RNAs in plastids affects RNA interference efficiency in the Colorado potato beetle
Source: J Exp Bot. 2020 Jan 6;71(9):2670–7. doi: 10.1093/jxb/eraa001 (PMC7210753; doi:10.1093/jxb/eraa001)
Supplement: eraa001_suppl_supplementary_figures_S1_S2_tables_S1_S2 [file eraa001_suppl_supplementary_figures_s1_s2_tables_s1_s2.pdf]

Supplementary Information

Length-dependent accumulation of double-stranded RNAs in plastids affects RNA interference efficiency in the Colorado potato beetle

Wanwan He[1], Wenbo Xu[1], Letian Xu[1], Kaiyun Fu[4], Wenchao Guo[3], Ralph Bock[1,2], Jiang Zhang[1*]

[1] State Key Laboratory of Biocatalysis and Enzyme Engineering, School of Life Sciences, Hubei University, Wuhan 430062, China.

[2] Max-Planck-Institut für Molekulare Pflanzenphysiologie, Am Mühlenberg 1, D-14476 Potsdam-Golm, Germany.

[3] Institute of Microbial Application, Xinjiang Agricultural Academy of Sciences, Urumqi, China

[4] Institute of Plant Protection, Xinjiang Academy of Agricultural Sciences, Urumqi, China

[*]*Corresponding author:* Zhang, J. (zhangjiang@hubu.edu.cn);

This PDF file includes:

Figures S1 to S2

Tables S1 to S2

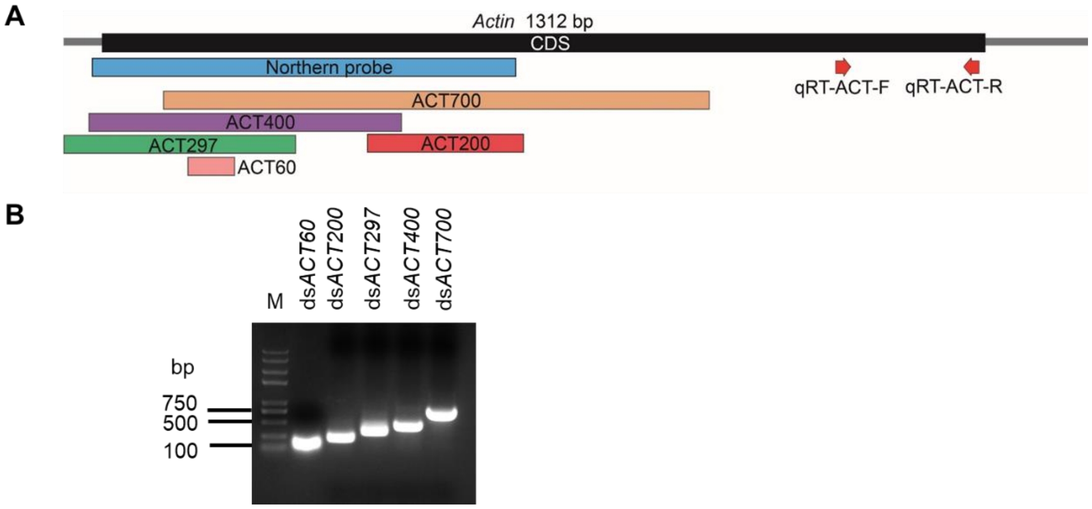

Fig. S1. *In vitro* synthesis of ds*ACT*s of different lengths. (**A**) Location of the different fragments for ds*ACT* synthesis within the *β-Actin* gene. The probe used for northern blot analysis of dsRNA expression in transplastomic potato plants is represented as a blue bar. The binding sites of primers for quantitative PCR analysis of *β-Actin* expression are indicated as red arrows. CDS: coding sequence. (**B**) The synthesized ds*ACTs* and their integrity were analyzed by electrophoresis in a 1% agarose gel. M, Trans 2K plusII DNA marker.

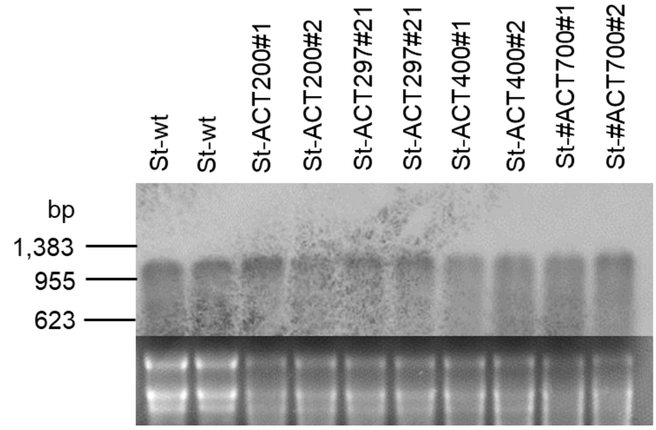

Fig. S2. Analysis of *psbA* transcript accumulation by northern blotting. 5 µg of total cellular RNA were loaded in each lane. The ethidium bromide-stained gel prior to blotting is shown below the blot.

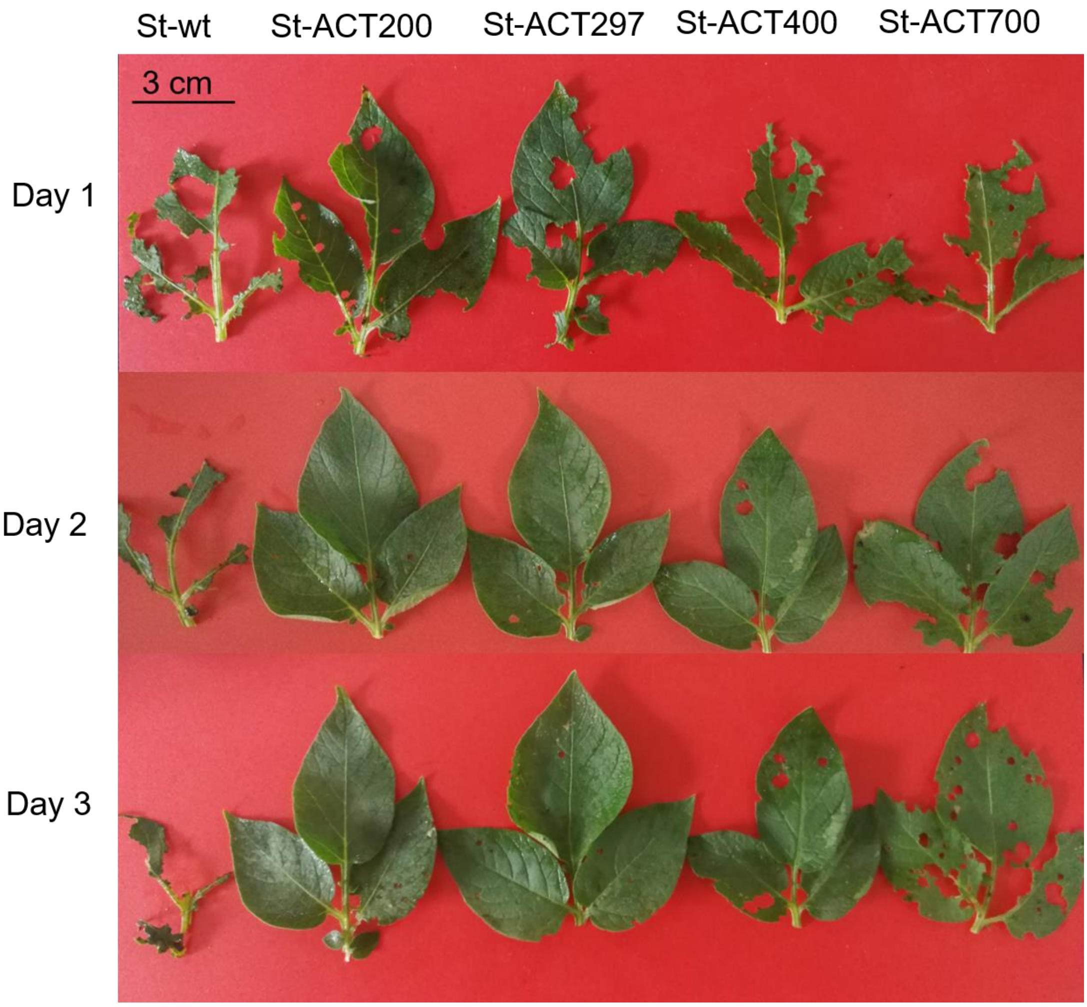

Fig. S3. Leaves of wide-type potato plants and transplastomic plants expressing different length of ds*ACTs* consumed by third-instar CPB larvae at the indicated time points. Note that less leaf material is consumed by CPB larvae with decreasing size of the ds*ACT* expressed in plastids.

Table S1. Summary of dsRNA accumulation levels in transplastomic lines expressing different dsRNA constructs (Zhang *et al*., 2015).

**Target gene**	***ACT***	***SHR***	***ACT+SHR***
dsRNA length (bp)	297	220	517
dsRNA GC content	52.19%	43.18%	48.36%
dsRNA accumulation level (% of total RNA)	0.4%	0.05%	0.1%
Mortality (at day 5)	100%	40%	16%

Table S2. List of oligonucleotides used in this study. Recognition sequences of introduced restriction sites are underlined.

Oligonucleotide	Sequence (5′-3′)	Description and Use
T7act60Fwd	GGATCCTAATACGACTCACTATAGGTCGCCCAAGGCATCAAGGAGTC	forward primer for amplification of the *ACT*60 fragment; introducing the T7 promoter sequence; for *in vitro* dsRNA synthesis
T7act60Rev	GGATCCTAATACGACTCACTATAGGTCTCCTACGTATGAGTCCTTTTG	reverse primer for amplification of the *ACT*60 fragment; introducing the T7 promoter sequence; for *in vitro* dsRNA synthesis
T7act200Fwd	GGATCCTAATACGACTCACTATAGGAAGCCAACAGGGAGAAGATG	forward primer for amplification of the *ACT*200 fragment; introducing the T7 promoter sequence; for *in vitro* dsRNA synthesis
T7act200Rev	GGATCCTAATACGACTCACTATAGGGTCCAAACGGAGGATGGCG	reverse primer for amplification of the *ACT*200 fragment; introducing the T7 promoter sequence; for *in vitro* dsRNA synthesis
T7act297Fwd	GGATCCTAATACGACTCACTATAGGGCACGAGGTTTTTCTGTCTAGT	forward primer for amplification of the *ACT*297 fragment; introducing the T7 promoter sequence; for *in vitro* dsRNA synthesis
T7act297Rev	GGATCCTAATACGACTCACTATAGGATGTCATCCCAGTTGGTG	reverse primer for amplification of the *ACT*297 fragment; introducing the T7 promoter sequence; for *in vitro* dsRNA synthesis
T7act400Fwd	GGATCCTAATACGACTCACTATAGGTCCAACCTCAAAAGACAAC	forward primer for amplification of the *ACT*400 fragment; introducing the T7 promoter sequence; for *in vitro* dsRNA synthesis
T7act400Rev	GGATCCTAATACGACTCACTATAGGGTCTCAAACATGATTTGGGTC	reverse primer for amplification of the *ACT*400 fragment; introducing the T7 promoter sequence; for *in vitro* dsRNA synthesis
T7act700Fwd	GGATCCTAATACGACTCACTATAGGCACCCCGTGCCGTCTTCC	forward primer for amplification of the *ACT*700 fragment; introducing the T7 promoter sequence; for *in vitro* dsRNA synthesis
T7act700Rev	GGATCCTAATACGACTCACTATAGGTGGGCAACGGAACCTCTC	reverse primer for amplification of the *ACT*700 fragment; introducing the T7 promoter sequence; for *in vitro*

		dsRNA synthesis
act200-F	AAGCCAACAGGGAGAAGATG	forward primer for amplification of the *ACT*200 fragment; for *in vitro* ssRNA synthesis
act200(Sbf I)-F	CTTGGATACGCATGCCTGCAGGAAGCCAACAGGGAGAAGATG	forward primer for amplification of the *ACT*200 fragment; introducing an SbfI site
act200(SacI)-R	ATACGAAGCGCTTGGATACGAGCTCGTCCAAACGGAGGATGGCG	reverse primer for amplification of the *ACT*200 fragment; introducing a SacI site
act400(SbfI)-F	CTTGGATACGCATGCCTGCAGGTCCAACCTCAAAAGACAAC	forward primer for amplification of the *ACT*400 fragment; introducing an SbfI site
act400(SacI)-R	ATACGAAGCGCTTGGATACGAGCTCGTCTCAAACATGATTTGGGTC	reverse primer for amplification of the *ACT*400 fragment; introducing a SacI site
act700(SbfI)-F	CTTGGATACGCATGCCTGCAGGCACCCCGTGCCGTCTTCC	forward primer for amplification of the *ACT*700 fragment; introducing an SbfI site
act700(SacI)-R	ATACGAAGCGCTTGGATACGAGCTCTGGGCAACGGAACCTCTC	reverse primer for amplification of the *ACT*700 fragment; introducing a SacI site
qRT-ACT-F	TGCAGAAGGAAATCACCGCT	forward primers for qRT-PCR analysis of *ACT* expression
qRT-ACT-R	CACTTGCGGTGAACGATTCC	reverse primers for qRT-PCR analysis of *ACT* expression
qRT-RP18-F	TAGAATCCTCAAAGCAGGTGGCGA	forward primers for qRT-PCR analysis of *RP18* expression (as reference gene)
qRT-RP18-R	AGCTGGACCAAAGTGTTTCACTGC	reverse primers for qRT-PCR analysis of *RP18* expression (as reference gene)
psbZ-S-F	GTGCGAATCCACCGGTCGATCTA	forward primer for probe synthesis for Southern blots
psbZ-S-R	AAGTAGCAATTAATGCAAAAACA	reverse primer for probe synthesis for Southern blots
actin-N-F	CAACCTCAAAAGACAACATG	forward primer for probe synthesis for northern blots analysis of *ACT* expression
actin-N-R	GGAGGATGGCGTGGGGAAGAGC	reverse primer for probe synthesis for northern blots analysis of *ACT* expression
psbA-N-F	ATGACTGCAATTTTAGAGA	forward primer for probe synthesis

	GACGCGAA	for northern blots analysis of potato *psbA* expression
psbA-N-R	GAGATTCCTAGAGGCATACCATCAGAAAA	reverse primer for probe synthesis for northern blots analysis of potato *psbA* expression
